# Development and validation of multivariate models integrating preoperative clinicopathological and radiographic findings to predict HER2 status in gastric cancer

**DOI:** 10.1038/s41598-022-18433-z

**Published:** 2022-08-19

**Authors:** Mengying Xu, Song Liu, Lin Li, Xiangmei Qiao, Changfeng Ji, Lingyu Tan, Zhengyang Zhou

**Affiliations:** 1 Department of Radiology, Nanjing Drum Tower Hospital, The Affiliated Hospital of Nanjing University Medical School, No.321, Zhongshan Road, Nanjing, 210008 Jiangsu China; 2 Department of Pathology, Nanjing Drum Tower Hospital, The Affiliated Hospital of Nanjing University Medical School, Nanjing, 210008 China; 3 Department of Ultrasound, Nanjing Drum Tower Hospital Clinical College of Nanjing Medical University, Nanjing, 210008 China

**Keywords:** Oncology, Cancer, Gastrointestinal cancer, Gastric cancer

## Abstract

The combination of trastuzumab and chemotherapy is recommended as first-line therapy for patients with human epidermal growth factor receptor 2 (HER2) positive advanced gastric cancers (GCs). Successful trastuzumab-induced targeted therapy should be based on the assessment of HER2 overexpression. This study aimed to evaluate the feasibility of multivariate models based on hematological parameters, endoscopic biopsy, and computed tomography (CT) findings for assessing HER2 overexpression in GC. This retrospective study included 183 patients with GC, and they were divided into primary (n = 137) and validation (n = 46) cohorts at a ratio of 3:1. Hematological parameters, endoscopic biopsy, CT morphological characteristics, and CT value-related and texture parameters of all patients were collected and analyzed. The mean corpuscular hemoglobin concentration value, morphological type, 3 CT value-related parameters, and 22 texture parameters in three contrast-enhanced phases differed significantly between the two groups (all *p* < 0.05). Multivariate models based on the regression analysis and support vector machine algorithm achieved areas under the curve of 0.818 and 0.879 in the primary cohort, respectively. The combination of hematological parameters, CT morphological characteristics, CT value-related and texture parameters could predict HER2 overexpression in GCs with satisfactory diagnostic efficiency. The decision curve analysis confirmed the clinical utility.

## Introduction

As a common malignant tumor of the gastrointestinal tract, gastric cancer (GC) is the fifth common tumor as well as the third cause of cancer death worldwide^1^. Some patients diagnosed with advanced gastric cancer require multidisciplinary treatment, such as combination of chemotherapy and targeted therapy^2^. According to the National Comprehensive Cancer Network (NCCN) Guidelines, the combination of trastuzumab and chemotherapy is recommended as first-line therapy for patients with human epidermal growth factor receptor 2 (HER2) positive advanced GCs, which has achieved favorable results^3^. However, successful trastuzumab-induced targeted therapy should be based on the assessment of HER2 overexpression^3^. Therefore, it is crucial to accurately evaluate HER2 status. In the current clinical practice, the overexpression of HER2 in GC is mainly tested by immunohistochemistry (IHC) based on endoscopic biopsy and surgical specimen^4^. Moreover, samples scored as IHC 2 + (equivocal) should be additionally detected by fluorescence in situ hybridization (FISH)^3,4^. However, endoscopic biopsy is invasive and possibly biased due to the sampling error^5^.

With the development of imaging tools, its application in assessing HER2 status is increasing. A previous study showed that the apparent diffusion coefficient value of magnetic resonance (MR) imaging could reflect the expression of HER2 in GC^6^. In addition, another study also indicated that the 18F-fluorodeoxyglucose positron emission tomography/computed tomography (18F-FDG PET/CT) scans may help to predict HER2 status in GC^7^. However, MR examination has longer acquisition time and encounters more artefacts, and PET-CT scan is relatively expensive and is accompanied by higher ionizing radiation^8,9^.

Contrast-enhanced CT is the most common imaging modality to evaluate GC. In addition, CT radiomics and texture analysis are increasingly applied in the histopathological assessment of GC^10–13^. Recent studies also demonstrated that CT radiomics can be used to assess the status of HER2 in GC. Li Y et al. mentioned that a nomogram based on CT radiomics and carcinoembryonic antigen (CEA) could predict HER2 status in GCs^14^. However, those above studies mainly focused on radiomics, part of them used CEA. Clinicopathological information, including hematological parameters and endoscopic biopsy, were still not fully used. In addition, CT morphological characteristics, and CT value-related and texture parameters can also be obtained from CT images. If the above information can be effectively used, the diagnostic performance to evaluate HER2 overexpression in GC might be further improved.

Consequently, this study aimed to evaluate the feasibility of multivariate models based on hematological parameters, endoscopic biopsy, CT morphological characteristics, and CT value-related and texture parameters for predicting HER2 overexpression in GC.

## Methods

The Ethical Committee of Nanjing Drum Tower Hospital approved this retrospective study and waived the requirement for informed consent. All methods were performed in accordance with relevant named guidelines and regulations.

### Patients

**Figure 1:**
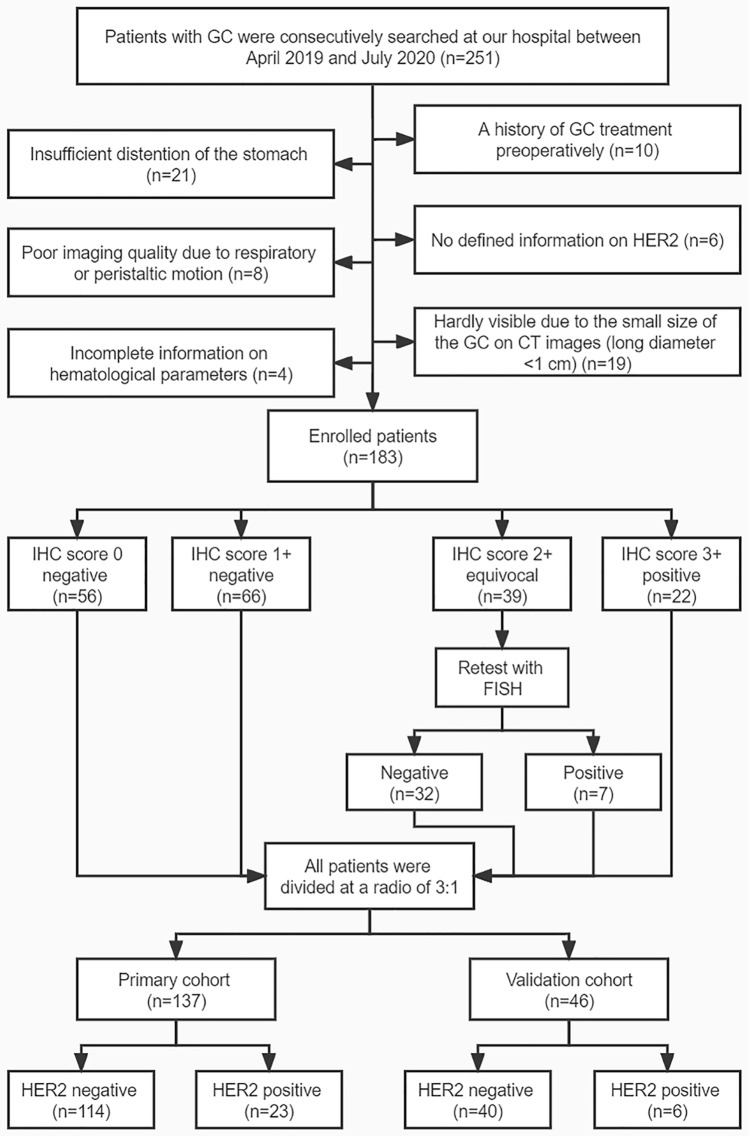
The flowchart of the enrolled patients in this study. *GC* gastric cancer, *CT* computed tomography, *HER2* human epidermal growth factor receptor 2, *IHC* immunohistochemistry, *FISH* fluorescence in situ hybridization.

From April 2019 to July 2020, two hundred and fifty-one consecutive patients with GC confirmed by histopathologic analysis were identified by searching radiologic image archives of our hospital. The inclusion criteria were: (1) pathological confirmation of GC postoperatively and (2) availability of endoscopic biopsy and abdominal contrast-enhanced CT within 2 weeks before surgery. The exclusion criteria were described in Fig. 1 and Supplementary Material.

**Figure 2:**
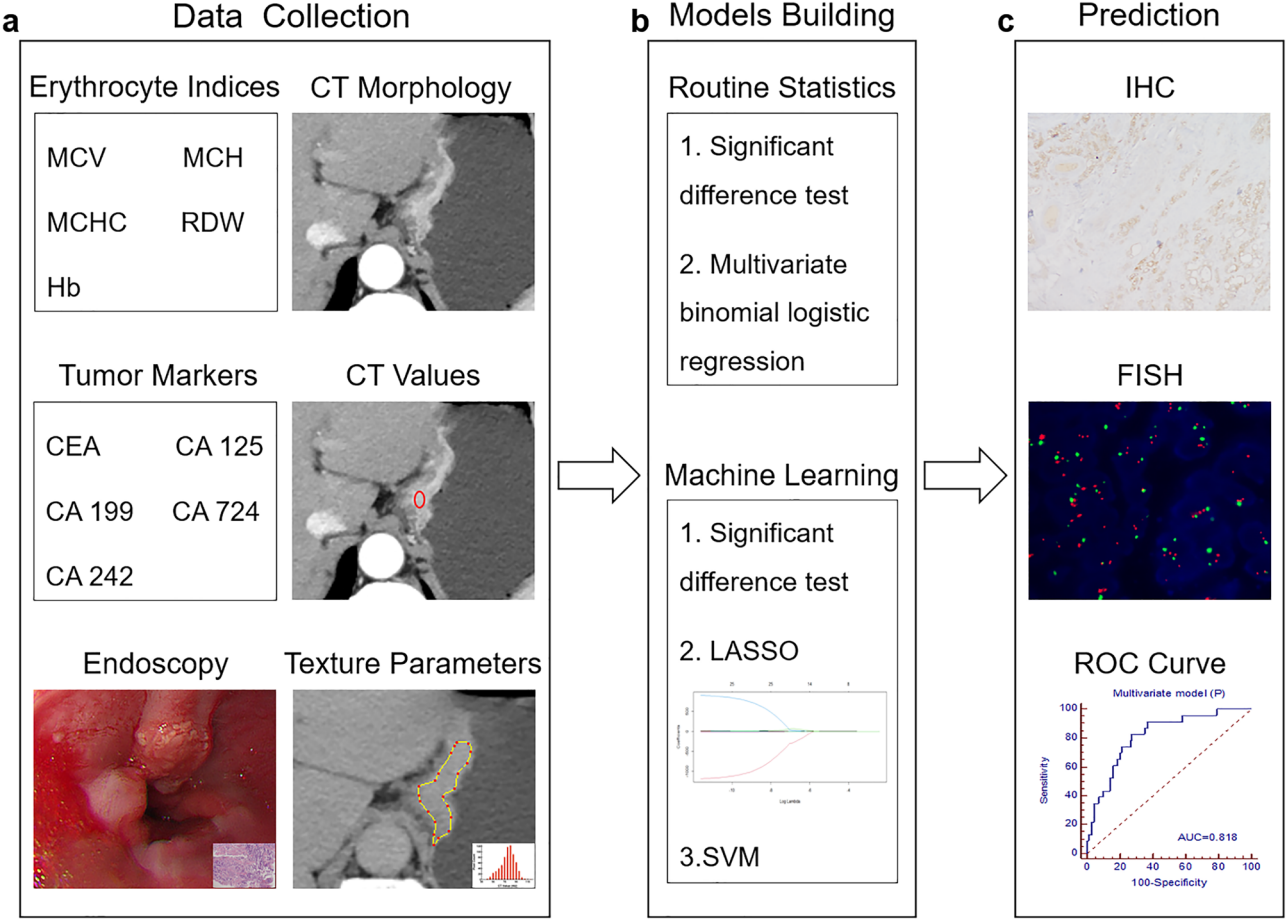
The workflow of this study. (**a**) Erythrocyte indices, tumor markers, differentiation degree based on biopsy, CT morphological characteristics, CT value-related and texture parameters were extracted. (**b**) Multivariate models were built based on binomial logistic regression and machine learning algorithm. (**c**) The overexpression of human epidermal growth factor receptor 2 (HER2) in gastric cancer was tested by IHC based on surgical specimens, samples scored as IHC 2 + (equivocal) were additionally detected by FISH. Diagnostic performance for predicting HER2 status was obtained by ROC curve analysis. *MCV* mean corpuscular volume, *MCH* mean corpuscular hemoglobin, *MCHC* mean corpuscular hemoglobin concentration, *RDW* red cell distribution width, *Hb* hemoglobin, *CEA* carcinoembryonic antigen, *CA* carbohydrate antigen, *CT* computed tomography, *LASSO* least absolute shrinkage and selection operator, *SVM* support vector machine, *IHC* immunohistochemistry, *FISH* fluorescence in situ hybridization, *ROC* receiver operating characteristic.

Finally, one hundred and eighty-three patients (male 128; female 55; median age 64 years; age range 30–86 years) conformed to the criteria. Patients were divided into primary cohort (n = 137) and validation cohort (n = 46) at a ratio of 3:1 according to the time of surgery. The flow chart of patient selection process is shown in Fig. 1. The demographic data and histopathological information of patients in the primary and validation cohorts are summarized in Table 1 and Supplementary Table S1, respectively. The overall framework of this study is depicted in Fig. 2. In addition, we added an extra cohort consisting of intestinal type GCs with 84 patients.

**Table 1:** Demographic data and histopathological information in the primary cohort.

Characteristics	HER2 negative (n = 114)	HER2 positive (n = 23)	*p*
**Demographic data**			
**Gender**			0.481
Male	81	18	
Female	33	5	
**Age (year)**			0.092
< 60	46	5	
≥ 60	68	18	
**Postoperative histopathological information**			
**Major location**			0.008*
Cardia	24	12	
Body	44	6	
Antrum	46	5	
**T stages**			0.491
1	6	2	
2	20	5	
3	59	13	
4	29	3	
**N stages**			0.334
N0	33	9	
N1–3	81	14	
**Lauren classification**^**a**^			0.003*
Intestinal type	44	18	
Diffuse type	32	2	
Mixed type	37	3	
**Lymphovascular invasion**			0.842
Absent	47	10	
Present	67	13	
**Neural invasion**			0.050
Absent	31	11	
Present	83	12	

*HER2* human epidermal growth factor receptor 2. ^a^Not applicable for one patient; *p < 0.05 with chi-square test or Fisher's exact test (n < 5).

### Hematological tests

Laboratory factors, including preoperative hemoglobin (Hb), serum mean corpuscular volume (MCV), mean corpuscular hemoglobin (MCH), mean corpuscular hemoglobin concentration (MCHC), red cell distribution width (RDW), CEA, carbohydrate antigen (CA) 125, CA199, CA724, and CA242, were obtained within 2 weeks prior to surgery.

### Endoscopic biopsy

The histological differentiation information based on preoperative endoscopic biopsy was examined and recorded retrospectively according to the WHO Classification of Tumours of the Digestive System (2019 version)^15^.

### CT image acquisition

Contrast-enhanced abdominal CT was performed using a 64-channel scanner (uCT 780, United Imaging). The details of patient preparation and imaging acquisition were described in Supplementary Material.

### Image analysis

#### CT morphological characteristics

Readers one and two (with 5 and 7 years of experience in the diagnosis of abdominal imaging, respectively) who were blinded to clinicopathological information except for the general tumor location, assessed the morphological characteristics of each lesion on transverse CT images independently, and their results were used to evaluate interobserver reliability. The third reader (with twenty years of experience in abdominal imaging) confirmed their inconsistent opinions as the final result. CT morphological characteristics were as follows: (1) infiltrative growth (absent, present): defined as unclear border between the lesion and the normal gastric wall; (2) ulceration (absent, present); (3) adjacent adipose tissue stains (absent, present); (4) mucosal line status (interruption, thickening); (5) morphological type (thickening type, mass type); (6) linitis plastica (absent, present); and (7) lymphadenectasis (absent, present): defined as a short-axis diameter of the regional LN over 1 cm in the upper abdomen^16^.

#### CT value-related parameters

The oval regions of interest (ROIs) were drawn in the same region with same shape and size in the four phases CT images. ROIs were drawn to encompass the area of greatest enhancement on the maximal section by reader one. The mean size of tumor ROIs was 36.75 mm^2^, and the range was 6.60–156.90 mm^2^. The mean CT attenuation values of the tumor in the non-enhanced (N), arterial phase (AP), portal phase (PP), and delayed phase (DP) were recorded as N value mean, AP value mean, PP value mean, and DP value mean, respectively, as well as the maximum and minimum CT values. With the N, AP, PP, and DP value means as the references, post-contrast tumorous attenuation differences (Δmean A–N, Δmean P–N, Δmean D–N, Δmean P–A, Δmean D–A, and Δmean D–P) were calculated^16^. CT value-related parameters extracted from ROIs delineated by reader one were used to predict HER2 status. Reader two repeated the above procedure to determine interobserver reproducibility.

#### CT texture analysis

The three-phase enhanced CT images were uploaded into in-house software (Image Analyzer 2.0). All the images were reviewed by reader one. Polygonal ROIs in the arterial phase (mean size 401.63 mm^2^; range 34.80–2442.87 mm^2^), portal phase (mean size 363.47 mm^2^; range 41.12–2129.96 mm^2^), and delayed phase (mean size, 406.32 mm^2^; range 67.29–1891.79 mm^2^) CT images were manually segmented along the tumor contour on the largest cross-section (Supplementary Fig. S1). The normal gastric wall tissue and the gastric cavity contents were avoided. The details and definitions of generated CT texture parameters were listed in Supplementary Table S2. Texture parameters derived from ROIs delineated by reader one were used to predict HER2 status. Reader two repeated the above procedure to determine interobserver reproducibility.

### Development, performance, and validation of multivariate models

On the one hand, start with the statistically significant (*p* < 0.05) variables in univariate analysis, multivariate binomial logistic regression analysis based on a backward elimination process was used to build the multivariate model in the primary cohort. The Hosmer–Lemeshow test was used to measure the goodness of fit. The model developed was also applied to the validation cohort and the extra cohort. The diagnostic performance of established model was evaluated with receiver operating characteristic (ROC) curve analysis. In addition, to evaluate the clinical usefulness of the multivariate model, a decision curve analysis (DCA) was plotted by demonstrating the net benefits graphically for a range of threshold probabilities in the validation cohort.

On the other hand, the statistically significant (*p* < 0.05) variables in the primary cohort were fed into the least absolute shrinkage and selection operator (LASSO) for dimension reduction. Then, the support vector machine (SVM) classifier with fivefold cross-validation was adopted to establish another multivariate model, and the model developed was also applied to the validation cohort.

### HER2 IHC and FISH method

The expression of HER2 status based on surgical specimen was tested by IHC and was classified as score 0 (IHC negative), score 1+ (IHC negative), score 2+ (IHC equivocal), and score 3+ (IHC positive)^4,17^. For equivocal cases at IHC (score 2+), additional FISH test was applied to assess HER2 overexpression in this study. Then, all patients were divided into two groups: HER2-negative (IHC 0, IHC 1+, IHC 2+ plus FISH negative) and HER2-positive (IHC 3+, IHC 2+ plus FISH positive). The details of IHC and FISH method were described in Supplementary Material.

### Statistical analysis

The differences between HER2-negative and HER2-positive groups in categorical variables, including demographic data, endoscopic biopsy, and morphological characteristics, were analyzed using the chi-square or Fisher's exact test (n < 5). And the differences of quantitative variables, including hematological parameters, CT value-related parameters, and CT texture parameters, were assessed by the Mann–Whitney U test after using the Shapiro–Wilk test for normality analysis. ROC curve analysis was performed, and the area under the ROC curve (AUC), diagnostic sensitivity, specificity, positive predictive value (PPV), negative predictive value (NPV), and accuracy were calculated. The cutoff value was established by calculating the largest Youden index (Youden index = sensitivity + specificity − 1). The kappa statistics was applied to evaluate the interobserver consistency, and the interobserver agreement of the CT parameters extracted by the two radiologists was estimated with intraclass correlation coefficient (ICC) (0.000–0.200: poor agreement; 0.201–0.400: fair agreement; 0.401–0.600: moderate agreement; 0.601–0.800: good agreement; 0.801–1.000: excellent agreement). Statistical analyses were performed using SPSS (version 22.0 for Microsoft Windows × 64, SPSS), MedCalc Statistical Software (version 11.4.2.0 MedCalc Software bvba; http://www.medcalc.org; 2011), and R software package (version 3.5.2: http://www.Rproject.org). Two-sided *p* value < 0.05 was considered significant.

## Results

### Qualitative analysis

#### Endoscopic biopsy

The differentiation degree based on endoscopic biopsy showed no significant difference between HER2-negative and HER2-positive groups in the primary cohort (*p* = 0.063, Table 2).

**Table 2:** Univariate analysis of endoscopic biopsy and morphological characteristics in the primary cohort.

Characteristics	HER2 negative (n = 114)	HER2 positive (n = 23)	*p*
**Endoscopic biopsy**			
**Differentiation degree**			0.063
Poor	82	12	
Moderate and well	32	11	
**Morphological characteristics**			
**Infiltrative growth**			0.092
Absent	68	18	
Present	46	5	
**Ulceration**			0.226
Absent	45	6	
Present	69	17	
**Adjacent adipose tissue stains**			0.446
Absent	74	13	
Present	40	10	
**Mucosal line status**			0.174
Interruption	47	6	
Thickening	67	17	
**Morphological type**			0.013*
Thickening type	33	1	
Mass type	81	22	
**Linitis plastica**			0.351
Absent	106	23	
Present	8	0	
**Lymphadenectasis**			0.051
Absent	100	16	
Present	14	7	

*HER2* human epidermal growth factor receptor 2; *p < 0.05 with chi-square test or Fisher's exact test (n < 5).

#### CT morphological characteristics

Table 2 summarizes the results of CT morphological characteristics between HER2-negative and HER2-positive groups in the primary cohort. The morphological type differed significantly between the two groups in the primary cohort (*p* = 0.013, Table 2).

### Quantitative analysis

#### Blood sample analysis

The univariate analysis for preoperative hematological parameters between HER2-negative and HER2-positive groups in the primary cohort are listed in Table 3. The MCHC value was significantly different between the two groups (*p* = 0.018, Table 3), and the corresponding AUC was 0.657 (Supplementary Table S3). There were no significant differences in tumor markers between HER2-negative and HER2-positive groups in the primary cohort (all *p* > 0.05).

**Table 3:** Statistical description and univariate analysis in the primary cohort.

Parameters	HER2 negative (n = 114)	HER2 positive (n = 23)	*p*
**Hematological parameter**			
MCHC (g/L)	337.00 (328.00, 344.00)	326.00 (321.00, 340.00)	0.018*
**CT value-related parameters**			
DP value mean (HU)	81.44 (71.33, 92.87)	74,85 (68.05, 81.29)	0.018*
DP value min (HU)	63.50 (50.75, 75.25)	56.00 (47.00, 61.00)	0.030*
Δmean D–N (HU)	41.52 (30.93, 52.58)	28.93 (22.94, 38.61)	0.003*
**Texture parameters (AP)**			
Histogram width (HU)	47.00 (39.00, 55.25)	39.00 (35.00, 53.00)	0.046*
**Texture parameters (PP)**			
75th percentile (HU)	97.00 (88.00, 108.50)	89.00 (82.00, 97.00)	0.049*
90th percentile (HU)	105.50 (95.00, 116.25)	98.00 (87.00, 106.00)	0.037*
Kurtosis	2.83 (2.57, 3.11)	3.02 (2.79, 3.27)	0.030*
Entropy	3.91 (3.71, 4.08)	3.80 (3.63, 3.97)	0.040*
Histogram width (HU)	35.50 (29.00, 43.00)	30.00 (26.00, 38.00)	0.045*
Entropy GLCM 10	6.78 (6.39, 7.19)	6.61 (6.23, 6.87)	0.031*
Entropy GLCM 13	6.52 (6.17, 6.88)	6.26 (6.07, 6.65)	0.040*
Energy GLCM 10^a^	11.75 (8.77, 15.65)	13.90 (11.47, 17.66)	0.027*
Energy GLCM 11^a^	13.01 (10.64, 17.58)	15.76 (12.77, 18.91)	0.046*
Energy GLCM 13^a^	14.49 (11.07, 18.20)	17.45 (13.21, 20.76)	0.030*
Variance GLCM 10	11.30 (7.72, 16.17)	8.45 (5.73, 12.79)	0.047*
Variance GLCM 12	11.36 (7.69, 16.05)	8.48 (6.04, 13.03)	0.048*
**Texture parameters (DP)**			
Mean (HU)	80.80 (72.92, 89.53)	73.24 (66.86, 80.92)	0.006*
Mode (HU)	79.00 (72.75, 91.25)	72.00 (66.00, 80.00)	0.006*
Maximum (HU)	115.00 (104.75, 125.00)	105.00 (99.00, 115.00)	0.008*
5th percentile (HU)	60.00 (52.00, 72.25)	56.00 (49.00, 62.00)	0.033*
10th percentile (HU)	64.50 (57.00, 76.00)	60.00 (53.00, 66.00)	0.037*
25th percentile (HU)	72.00 (64.00, 82.25)	68.00 (60.00, 74.00)	0.019*
50th percentile (HU)	81.00 (73.00, 90.00)	73.00 (67.00, 80.00)	0.005*
75th percentile (HU)	89.00 (81.00, 97.00)	80.00 (74.00, 88.00)	0.004*
90th percentile (HU)	95.00 (88.00, 104.50)	87.00 (82.00, 94.00)	0.002*

The data are presented as median with (1st quartile, 3rd quartile); *HER2* human epidermal growth factor receptor 2, *MCHC* mean corpuscular hemoglobin concentration, *CT* computed tomography, *AP* arterial phase, *PP* portal phase, *DP* delayed phase, *GLCM* gray-level cooccurrence matrix; ^a^× 10^–3^; *p < 0.05 with Mann–Whitney U test.

#### CT value-related parameters

As shown in Table 3, there were significant differences in DP value mean, DP value min, and Δmean D–N (*p* = 0.018, 0.030, and 0.003, respectively), and the corresponding AUCs were 0.657, 0.644, and 0.695, respectively (Supplementary Table S3).

#### CT texture parameters

There were significant differences between the two groups in one parameter in arterial phase, twelve parameters in portal phase, and nine parameters in delayed phase, respectively (all *p* < 0.05, Table 3). And the corresponding AUCs ranged from 0.630 to 0.701 (Supplementary Table S3).

### Development, performance, and validation of multivariate models

#### Multivariate binomial logistic regression

**Figure 3:**
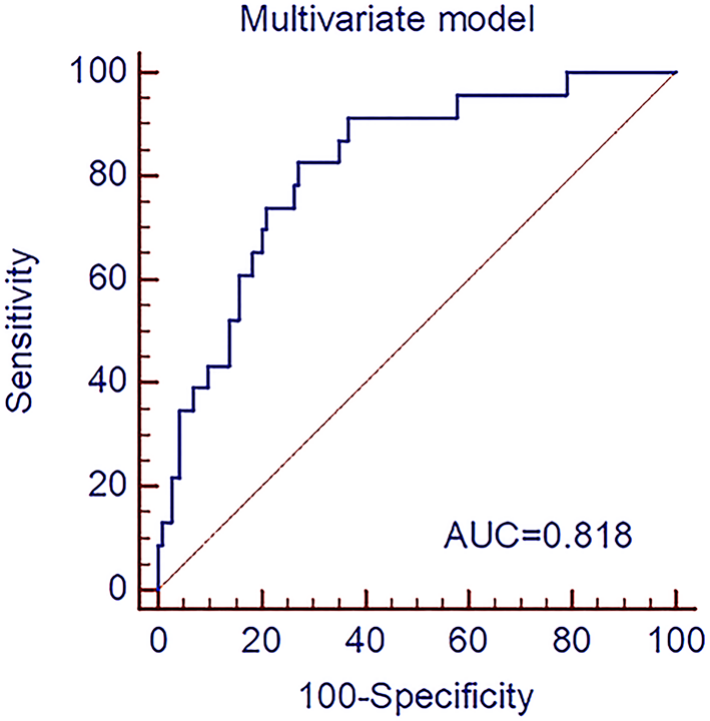
Receiver operating characteristic curve of multivariate model based on binomial logistic regression analysis for predicting human epidermal growth factor receptor 2 status of gastric cancer in the primary cohort. The AUC of the multivariate model was 0.818. *AUC* area under the receiver operating characteristic curve.

**Figure 4:**
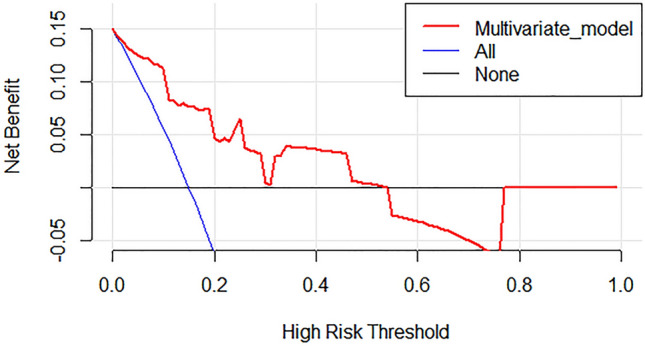
Decision curve analysis for the multivariate model based on regression analysis in the validation cohort. The y-axis indicates the net benefit; x-axis indicates threshold probability. The multivariate model (red line) had the highest net benefit compared with the simple diagnoses such as all HER2-positive GC patients (blue line) or all HER2-negative GC patients (black line) across the majority of the range of reasonable threshold probabilities at which a patient would be diagnosed as HER2-positive.

The best-performing model based on regression analysis for discriminating HER2-negative from HER2-positive groups in the primary cohort consisted of MCHC, morphological type, two texture parameters in portal phase, and three texture parameters in delayed phase (Table 4). The multivariate model had a predictive ability with a cutoff value of 0.19 (AUC = 0.818, *p* < 0.001), which yield a sensitivity, specificity, PPV, NPV, and accuracy of 82.6%, 72.8%, 38.0%, 95.4%, and 74.4%, respectively. The cutoff value of 0.19 was applied to test the predictive performance of the validation cohort, which yield a sensitivity, specificity, PPV, NPV, and accuracy of 66.7%, 87.5%, 44.4%, 94.6%, and 84.8%, respectively. The ROC curve of the primary cohort is shown in Fig. 3. The DCA for the multivariate model is plotted in Fig. 4.

**Table 4:** Multivariable binomial logistic regression results for predicting HER2 status in the primary cohort.

Parameters	Β level	S.E	Wald	*p*	OR (95% CI)
Morphological type	2.53	1.20	4.45	0.035	12.53 (10.18, 14.88)
MCHC	− 0.03	0.02	2.83	0.093	0.97 (0.94, 1.00)
Variance GLCM 10 (PP)	0.97	0.58	2.79	0.095	2.64 (1.50, 3.77)
Variance GLCM 12 (PP)	− 1.04	0.60	3.01	0.083	0.35 (− 0.82, 1.53)
10th percentile (DP)	0.21	0.12	3.22	0.073	1.24 (1.00, 1.47)
50th percentile (DP)	− 0.63	0.29	4.64	0.031	0.53 (− 0.04, 1.10)
75th percentile (DP)	0.35	0.20	3.16	0.075	1.42 (1.03, 1.80)

*S.E.* standard error, *OR* odds ratio, *CI* confidence interval, *MCHC* mean corpuscular hemoglobin concentration, *PP* portal phase, *DP* delayed phase, *GLCM* gray-level cooccurrence matrix.

In addition, to test whether the multivariate model could accurately distinguish HER2-negative and -positive GCs in intestinal type GCs, we used the cutoff value of 0.19 (the same as the logistic regression model developed in the primary cohort) to test the predictive performance of this extra cohort, which yield a sensitivity, specificity, PPV, NPV, and accuracy of 86.4%, 54.8%, 60.2%, 40.4%, and 91.9%, respectively.

#### Machine learning algorithms

**Figure 5:**
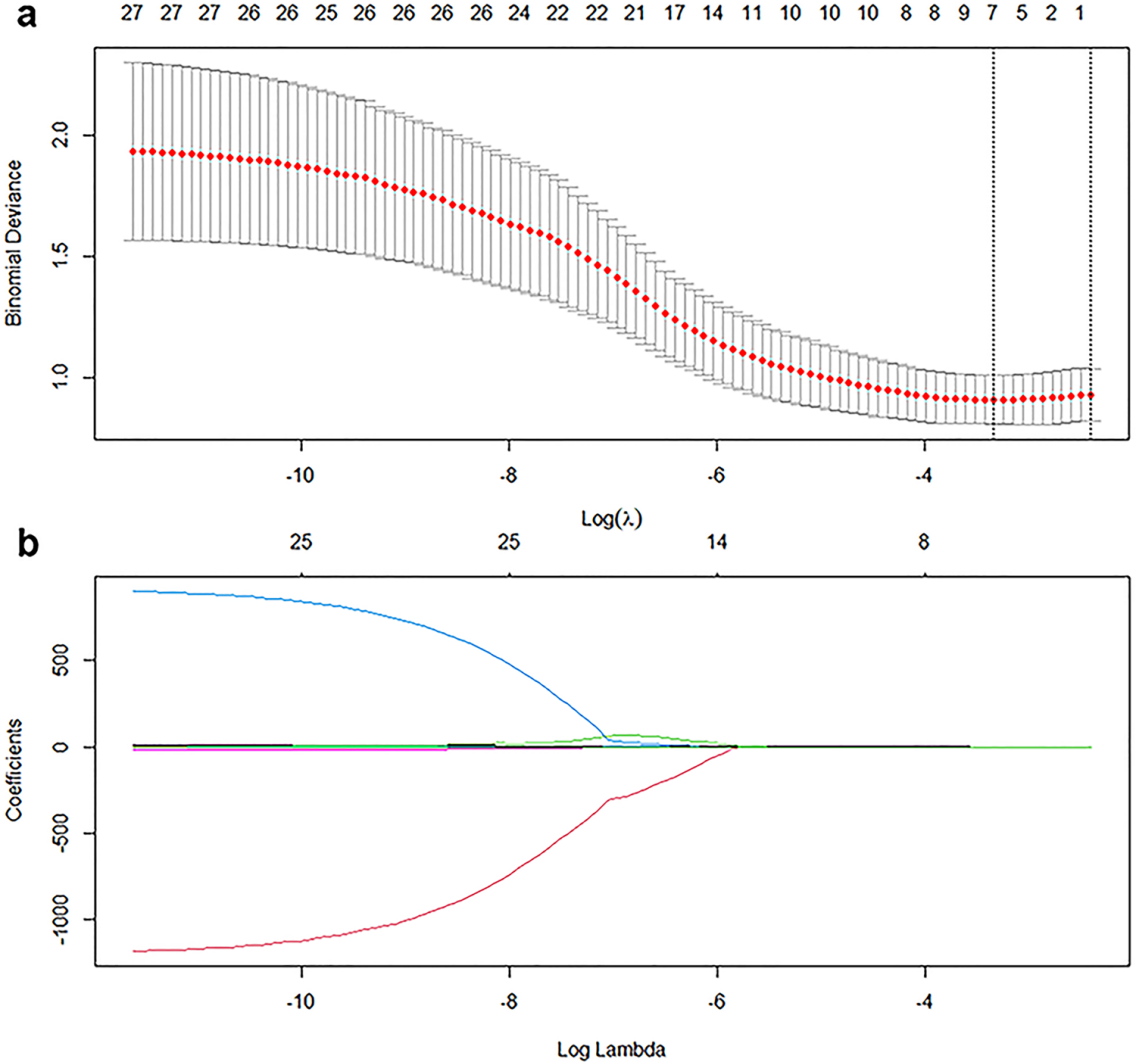
Feature selection was performed using the least absolute shrinkage and selection operator (LASSO) regression model. (**a**) Tuning parameter (λ) selection in the LASSO model used fivefold cross-validation via minimum criteria. Vertical lines were drawn at the optimal values using the minimum criteria and 1 standard error of the minimum criteria. The optimal λ value of 0.0356 with log (λ) = − 3.3359 was chosen. (**b**) LASSO coefficient profiles of the 27 selected features. A coefficient profile plot was generated versus the selected log (λ) value using fivefold cross-validation; seven selected features with nonzero coefficients were retained.

LASSO was performed to reduce the dimensionality of the features and to select optimal variables in the primary cohort (Fig. 5). Finally, MCHC, morphological type, Δmean D–N, histogram width in arterial phase, Entropy gray-level cooccurrence matrix (GLCM) 10 in portal phase, and mode, 90th percentile in delayed phase were integrated to build a multivariate model using the SVM algorithm in the primary cohort, which achieved an AUC of 0.879. The developed model was also applied to the validation cohort with an AUC of 0.921.

### Interobserver agreement

All CT morphological characteristics in the evaluation of GCs showed good to excellent interobserver agreement (κ = 0.652–0.903) (Supplementary Table S4). All CT value-related parameters (ICC = 0.699–0.950) and texture parameters based on arterial phase (ICC = 0.676–0.988) also showed good to excellent interobserver agreement (Supplementary Tables S5 and S6). For texture parameters based on portal and delayed phases, there were 23/35 and 29/35 parameters showed good to excellent interobserver agreement, respectively (Supplementary Tables S7 and S8).

## Discussion

In this current study, we investigated the utility of multivariate models integrating clinicopathological features and CT findings to predict HER2 status in GC preoperatively. The differentiation degree based on endoscopic biopsy, preoperative hematological parameters, 7 CT morphological characteristics, 18 CT value-related parameters, and 35 CT texture parameters in three contrast-enhanced phases were collected for building the multivariate models. There were significant differences in multiple features between HER2-negative and HER2-positive groups.

First, for CT morphological characteristics and conventional CT values, we found that thickening type lesions based on CT morphology were more common in HER2-negative GCs. It is also reported that most HER2-negative GCs tended to be diffuse types based on Lauren classification^18–20^. Tumor cells of diffuse type GCs are scattered in stomach wall and could not form obvious masses, which are more likely to display as thickening type on CT morphology. Accurate Lauren classification can only be accurately obtained by pathological evaluation of surgical specimens, while CT morphological characteristics are easy to evaluate preoperatively. In this study, CT value-related parameters based on the phase of 180 s delay, including DP value mean, DP value min, and Δmean D–N, were significantly higher in HER2-negative group in the primary cohort. It indicated that the enhancement degree in HER2-negative GCs were higher than in HER2-positive GCs in DP. Previous studies showed that HER2-negative GCs tended to be poorly differentiated and more aggressive^18,19^. Moreover, Tsurumaru et al. also reported that the CT values of undifferentiated type GCs were significantly higher than those of differentiated or mixed type in the delayed phase^21^. We reviewed relevant studies and found that CT parameters based on the phase of 180 s delay were widely used in the evaluation of hepatocellular carcinoma^22–24^. Sano et al. mentioned that the CT values based on the phase of 180 s delay were significantly different between hepatocellular carcinoma and intrahepatic cholangiocarcinoma^22^.

Second, for CT texture parameters, 22 parameters, including one parameter (histogram width) in AP, twelve parameters (75th–90th percentiles, kurtosis, entropy, histogram width, Entropy GLCM 10, 13, Energy GLCM 10, 11, 13, and Variance GLCM 10, 12) in PP, and nine parameters (mean, mode, maximum, and 5th–90th percentiles) in DP, differed significantly between HER2-negative and -positive groups. The values of percentiles, mean, and maximum reflect the enhancement degree of tumor. In this study, 75th–90th percentiles in PP, mean, maximum, and 5th–90th percentiles in DP were all higher in HER2-negative GCs. It indicated that the contrast media washed out slowly in HER2-negative GCs compared with HER2-positive GCs. Entropy and Entropy GLCM reflect the complexity of pixel distributions, Energy GLCM indicates the uniformity of pixel distributions, and Variance GLCM reflects the textural dispersion of the gray values around the mean^10,25–27^. Our study found that the values of entropy, Entropy GLCM (10, 13), and Variance GLCM (10, 12) were significantly higher in HER2-negative GCs, while the values of kurtosis and Energy GLCM (10, 11, 13) were significantly lower in HER2-negative GCs. It may because HER2-negative GCs tended to be poorly differentiated and more aggressive^18,19^, which resulted in heterogeneous pixel distributions of tumors. Moreover, five parameters, including Variance GLCM (10, 12) in PP and percentiles (10th, 50th, 75th) in DP, were retained after the backward elimination process using binomial logistic regression analysis. It indicates that texture parameters in PP and DP could better differentiate HER2-negative from HER2-positive GCs.

Third, for preoperative clinical information, the MCHC value differed significantly between HER2-negative and -positive groups in the primary cohort. Recently, a number of studies have reported that hematologic parameters, including Hb, MCV, MCH, MCHC and RDW, could be biomarkers for the diagnosis and prognosis of GCs^28–32^. Pietrzyk et al. found that blood indicators such as RDW could differentiate patients with GC from healthy individuals^32^. Moreover, Jomrich et al. reported that MCV and MCHC were significantly correlated with overall survival and disease-free survival in patients with gastroesophageal adenocarcinoma^31^. In this study, we found that the MCHC value was lower in HER2-positive group. HER2 overexpression is more common in intestinal type GCs^18–20^. Tumor cells of intestinal type are prone to aggregate and form obvious masses, which appears as mass type on CT morphology and is more likely to cause local ischemia and ulceration, leading to lower MCHC value. In addition, there were no significant differences in differentiation degree based on biopsy and tumor markers between HER2-negative and -positive groups in the primary cohort. Previous studies also analyzed the relationship between serum tumor markers and HER2 status in GC, however, their conclusions were controversial.

Furthermore, we developed a multivariate model based on binomial logistic regression analysis, which achieved an AUC of 0.818 in the primary cohort. DCA indicated the multivariate model based on regression analysis was clinically useful in this current study. Meanwhile, another multivariate model was developed based on the SVM algorithm, which achieved better performance in the primary cohort (AUC = 0.879). The developed multivariate models were also used in the validation cohort and achieved favorable performance. Wang et al. reported that radiomics based on AP and PP CT images could distinguish HER2-negative GCs with AUCs of 0.756 and 0.715, respectively^12^. Moreover, Li et al. mentioned that a nomogram based on CT radiomics and CEA could assess HER2 status in GCs with an AUC of 0.799^14^. In our study, the diagnostic efficiency was higher, it may because preoperative comprehensive information, including hematological parameters, morphological characteristics, CT value-related and texture parameters, was utilized and integrated. To test whether the multivariate model established in this study could accurately distinguish HER2-negative and -positive GCs in intestinal type GCs, we used the cutoff value of 0.19 (the same as the logistic regression model developed in the primary cohort) to test the predictive performance of this extra cohort, which yield a sensitivity, specificity, PPV, NPV, and accuracy of 86.4%, 54.8%, 60.2%, 40.4%, and 91.9%, respectively.

Certain limitations in our study are worthy of consideration. First, this was a retrospective study in a single center, and GCs without surgery were not included, which might have led to sample selection bias. Second, the number of HER2-positive GCs was relatively small in this study, multivariate models established in this study need to be further validated by multicenter large-scale studies as well as prospective studies. Third, the CT scan for all patients was performed using a single machine, thus multiple CT scanners need to be applied to validate the diagnostic performance. Fourth, texture features were extracted based on two-dimensional ROIs instead of three-dimensional volumes of interest, which might have lost longitudinal information. However, the application of two-dimensional ROIs was convenient in clinical practice. Therefore, prospective multicenter studies need to be conducted to solve the above problems.

In conclusion, the combination of hematological parameters, morphological characteristics, CT value-related and texture parameters could predict HER2 overexpression in GCs with satisfactory diagnostic efficiency.

## Supplementary Information

Supplementary Information.

## Data Availability

The datasets used and analyzed during the current study are available from the corresponding author on reasonable request.
