# Supplementary material for: Development and validation of multivariate models integrating preoperative clinicopathological and radiographic findings to predict HER2 status in gastric cancer
Source: Sci Rep. 2022 Aug 19;12:14177. doi: 10.1038/s41598-022-18433-z (PMC9391326; doi:10.1038/s41598-022-18433-z)
Supplement: Supplementary file 1 — Supplementary Information. [file 41598_2022_18433_MOESM1_ESM.pdf]

S1. The exclusion criteria

The exclusion criteria were as follows: (1) a history of GC treatment preoperatively (n=10); (2) insufficient distention of the stomach (n=21); (3) no defined information on HER2 (n=6); (4) poor imaging quality due to respiratory or peristaltic motion (n=8); (5) hardly visible due to the small size of GC on CT images (long diameter <1 cm) (n=19); and (6) incomplete information on hematological parameters (n=4).

S2. CT acquisition parameters

All patients were required to fast for no less than 6 hours and take 600-1000 mL of warm water orally prior to examination. All patients were in the supine position, and the scan covered the upper or the entire abdomen. CT images were obtained 40 s (arterial phase), 70 s (portal phase) and 180 s (delayed phase) after an infusion of 1.5 mL/kg of iodinated contrast agent (Omnipaque 350 mg I/mL, GE Healthcare) at a rate of 3 mL/s by a high-pressure syringe via the antecubital vein. The CT scan parameters were as follows: tube voltage 120 kV, tube current 150-250 mA, field of view 35-50 cm, matrix 512 × 512, rotation time 0.7 s, and pitch 1.0875. CT images were reconstructed with a 1-mm section thickness for multiplanar reconstruction and with a 5-mm section thickness for the measurement of CT values due to the signal-to-noise ratio.

S3. HER2 IHC and FISH method

All the patients underwent gastrectomy (either total or partial). All gastric specimens were processed according to standard pathological procedures. Immunohistochemical analysis using the primary rabbit antihuman HER2 polyclonal antibody (clone A0485, dilution: 1:2500, Dako) were performed on

paraffin-embedded gastric cancer specimens, which were cut at 4-μm in thickness. The details of IHC scoring standard in gastric cancer and showed as follows: 1) score 3+ (IHC positive): defined as strong complete or basolateral membranous staining in ≥10% of the neoplastic cells; 2) score 2+ (IHC equivocal): defined as moderate/weak complete or basolateral membranous staining in ≥10% of the cells; 3) score 1+ (IHC negative): defined as staining is weak or detected in only one part of the membrane in ≥10% of the cells; and 4) score 0 (IHC negative): defined as no membranous staining or staining of <10% of the tumor cells [1, 2].

For equivocal cases at IHC (score 2+), additional FISH test using locus-specific HER2 probe (190-kb Spectrum Orange directly labeled fluorescent DNA probe) and CEP17 probe (5.4-kb Spectrum Green directly labeled fluorescent DNA) was applied to assess the HER2 overexpression in this study. The details of FISH scoring standard in gastric cancer were showed as follows: 1) positive: defined as the HER2/CEP17 ratio ≥2; and 2) negative: defined as the HER2/CEP17 ratio <2 [2, 3].

Reference

1. Abrahao-Machado LF, Scapulatempo-Neto C. HER2 testing in gastric cancer: An update. *World J Gastroenterol*. **22,** 4619-4625(2016).

2. Hofmann M. et al. Assessment of a HER2 scoring system for gastric cancer: results from a validation study. *Histopathology*. **52,** 797-805(2008).

3. Li Y. et al. A CT-based radiomics nomogram for prediction of human epidermal growth factor receptor 2 status in patients with gastric cancer. *Chin J Cancer Res.* **32,** 62-71 (2020).

Table S1 Demographic data and histopathological information in the validation cohort

Characteristics	HER2 Negative (n=40)	HER2 Positive (n=6)	*p*
Demographic data			
Gender			0.071
Male	23	6	
Female	17	0	
Age (y)			0.647
<60	14	1	
≥60	26	5	
Postoperative histopathological information			
Major location			0.116
Cardia	11	1	
Body	16	5	
Antrum	13	0	
T stages			0.164
1	2	1	
2	4	0	
3	21	5	
4	13	0	
N stages			0.350
N0	11	3	
N1-3	29	3	
Lauren classification			0.850
Intestinal type	18	4	
Diffuse type	9	1	
Mixed type	13	1	
Lymphovascular invasion			0.667
Absent	20	2	
Present	20	4	
Neural invasion			0.598
Absent	8	2	
Present	32	4	

HER2, human epidermal growth factor receptor 2; *$p<0.05$ with chi-square test or Fisher's exact test (n<5).

Table S2 Details and definitions of CT texture parameters

Category	Textural feature	Definition
First-order features	Mean	mean
	Minimum	minimum
	Maximum	maximum
	5th-90th percentiles	n^{th} percentile grey-level intensity of a cumulative histogram
	Standard deviation	degree of dispersion of pixel gray level distributions
	Max frequency	peak value of a histogram
	Mode	gray level value that appears most frequently in a histogram
	Skewness	asymmetry of the pixel distribution
	Kurtosis	peakedness and tailedness of the histogram
	Entropy	randomness in the gray level values
	Histogram width	range of gray values in the ROI
Second-order features	Entropy GLCM	randomness of gray level values in the spatial distribution
	Energy GLCM	average of gray level values in the spatial distribution
	Inertia GLCM	variation in gray level intensities
	Variance GLCM	spread of spatial gray-level distribution

ROI, region of interest; GLCM, gray-level cooccurrence matrix.

Reference

1. Reinert CP, Krieg EM, Bösmüller H, Horger M. Mid-term response assessment in multiple myeloma using a texture analysis approach on dual energy-CT-derived bone marrow images - A proof of principle study. *Eur J Radiol*. **131,** 109214(2020).

2. Dieckmeyer M. et al. Gender-, Age- and Region-Specific Characterization of Vertebral Bone Microstructure Through Automated Segmentation and 3D Texture Analysis of Routine Abdominal CT. *Front Endocrinol (Lausanne)*. **12,** 792760(2022).

3**.** Lu Z. et al. Prediction of Clinical Pathologic Prognostic Factors for Rectal Adenocarcinoma: Volumetric Texture Analysis Based on Apparent Diffusion Coefficient Maps. *J Med Syst*. **43,** 331(2019).

Table S3 Diagnostic performance of hematological parameter, the CT value-related parameters, and texture parameters in the primary cohort

Parameters	Cutoff	Sensitivity	Specificity	AUC	Accuracy	*p*
Hematological parameters						
MCHC (g/L)	329.00	0.609	0.737	0.657	0.716	0.009*
CT value-related parameters						
DP value mean (HU)	84.12	0.870	0.447	0.657	0.518	0.006*
DP value min (HU)	61.00	0.783	0.553	0.644	0.592	0.010*
Δmean D-N (HU)	40.59	0.870	0.526	0.695	0.584	0.001*
Texture parameters (AP)						
Histogram width (HU)	40.00	0.609	0.649	0.632	0.642	0.045*
Texture parameters (PP)						
75th percentile (HU)	95.00	0.739	0.526	0.630	0.562	0.037*
90th percentile (HU)	106.00	0.870	0.483	0.638	0.548	0.025*
Kurtosis	2.97	0.652	0.623	0.644	0.628	0.008*
Entropy	3.99	0.870	0.386	0.636	0.467	0.019*
Histogram width (HU)	34.00	0.739	0.544	0.633	0.577	0.034*
Entropy GLCM 10	6.93	0.870	0.412	0.643	0.489	0.011*
Entropy GLCM 13	6.78	0.957	0.333	0.636	0.438	0.017*
Energy GLCM 10	11.40 [a]	0.826	0.474	0.646	0.533	0.007*
Energy GLCM 11	12.60 [a]	0.826	0.474	0.632	0.533	0.022*
Energy GLCM 13	12.10 [a]	0.957	0.342	0.643	0.445	0.013*
Variance GLCM 10	6.91	0.435	0.816	0.632	0.753	0.042*
Variance GLCM 12	6.75	0.435	0.807	0.631	0.745	0.034*
Texture parameters (DP)						
Mean (HU)	81.00	0.826	0.491	0.682	0.547	0.001*
Mode (HU)	76.00	0.739	0.623	0.682	0.642	0.001*
Maximum (HU)	119.00	0.957	0.404	0.677	0.497	0.001*
5th percentile (HU)	70.00	1.000	0.281	0.641	0.402	0.010*
10th percentile (HU)	66.00	0.826	0.465	0.638	0.526	0.011*
25th percentile (HU)	75.00	0.870	0.430	0.655	0.504	0.004*
50th percentile (HU)	80.00	0.783	0.509	0.684	0.555	0.001*
75th percentile (HU)	88.00	0.826	0.518	0.689	0.570	0.001*
90th percentile (HU)	87.00	0.565	0.781	0.701	0.745	<0.001*

AUC, area under the receiver operating characteristic (ROC) curve; HER2, human epidermal growth factor receptor 2; MCHC, mean corpuscular hemoglobin concentration; AP, arterial phase; PP, portal phase; DP, delayed phase; GLCM, gray-level cooccurrence matrix; [a], $\times 10^{-3}$; *p<0.05 with ROC curve analysis.

Table S4 Interobserver agreement for CT morphological characteristics

Characteristics	κ
Infiltrative growth	0.725
Ulceration	0.652
Adjacent adipose tissue stains	0.794
Mucosal line status	0.723
Morphological type	0.752
Linitis plastica	0.903
Lymphadenectasis	0.886

Table S5 Interobserver agreement for CT value-related parameters

Parameters	ICC	Parameters	ICC
N value mean	0.824	DP value mean	0.936
N value max	0.795	DP value max	0.911
N value min	0.699	DP value min	0.866
AP value mean	0.950	Δmean A-N	0.945
AP value max	0.940	Δmean P-N	0.899
AP value min	0.892	Δmean D-N	0.918
PP value mean	0.915	Δmean P-A	0.901
PP value max	0.886	Δmean D-A	0.940
PP value min	0.844	Δmean D-P	0.851

ICC, intraclass correlation coefficient; N, non-enhanced phase; AP, arterial phase; PP, portal phase; DP, delayed phase.

Table S6 Interobserver agreement for CT texture parameters based on arterial phase

Parameters	ICC	Parameters	ICC
Mean	0.971	Histogram width	0.861
Standard deviation	0.856	Entropy GLCM 10	0.832
Max frequency	0.977	Entropy GLCM 11	0.836
Mode	0.928	Entropy GLCM 12	0.820
Minimum	0.807	Entropy GLCM 13	0.828
Maximum	0.963	Energy GLCM 10	0.780
5th percentile	0.908	Energy GLCM 11	0.807
10th percentile	0.927	Energy GLCM 12	0.741
25th percentile	0.952	Energy GLCM 13	0.780
50th percentile	0.969	Inertia GLCM 10	0.810
75th percentile	0.982	Inertia GLCM 11	0.874
90th percentile	0.988	Inertia GLCM 12	0.847
Skewness	0.676	Inertia GLCM 13	0.796
Kurtosis	0.760	Variance GLCM 10	0.874
Entropy	0.824	Variance GLCM 11	0.866
Area	0.966	Variance GLCM 12	0.867
Max diameter	0.934	Variance GLCM 13	0.866
SsD low	0.850		

ICC, intraclass correlation coefficient; GLCM, gray-level cooccurrence matrix.

Table S7 Interobserver agreement for CT texture parameters based on portal phase

Parameters	ICC	Parameters	ICC
Mean	0.938	Histogram width	0.636
Standard deviation	0.608	Entropy GLCM 10	0.603
Max frequency	0.940	Entropy GLCM 11	0.611
Mode	0.857	Entropy GLCM 12	0.600
Minimum	0.698	Entropy GLCM 13	0.632
Maximum	0.916	Energy GLCM 10	0.544
5th percentile	0.838	Energy GLCM 11	0.562
10th percentile	0.871	Energy GLCM 12	0.538
25th percentile	0.913	Energy GLCM 13	0.600
50th percentile	0.936	Inertia GLCM 10	0.852
75th percentile	0.953	Inertia GLCM 11	0.802
90th percentile	0.961	Inertia GLCM 12	0.755
Skewness	0.522	Inertia GLCM 13	0.867
Kurtosis	0.473	Variance GLCM 10	0.593
Entropy	0.583	Variance GLCM 11	0.582
Area	0.879	Variance GLCM 12	0.584
Max diameter	0.871	Variance GLCM 13	0.591
SsD low	0.693		

ICC, intraclass correlation coefficient; GLCM, gray-level cooccurrence matrix.

Table S8 Interobserver agreement for CT texture parameters based on delayed phase

Parameters	ICC	Parameters	ICC
Mean	0.936	Histogram width	0.680
Standard deviation	0.678	Entropy GLCM 10	0.653
Max frequency	0.913	Entropy GLCM 11	0.659
Mode	0.902	Entropy GLCM 12	0.652
Minimum	0.618	Entropy GLCM 13	0.650
Maximum	0.915	Energy GLCM 10	0.381
5th percentile	0.866	Energy GLCM 11	0.385
10th percentile	0.895	Energy GLCM 12	0.416
25th percentile	0.921	Energy GLCM 13	0.459
50th percentile	0.939	Inertia GLCM 10	0.818
75th percentile	0.944	Inertia GLCM 11	0.828
90th percentile	0.943	Inertia GLCM 12	0.813
Skewness	0.492	Inertia GLCM 13	0.827
Kurtosis	0.318	Variance GLCM 10	0.715
Entropy	0.637	Variance GLCM 11	0.709
Area	0.819	Variance GLCM 12	0.715
Max diameter	0.853	Variance GLCM 13	0.716
SsD low	0.756		

ICC, intraclass correlation coefficient; GLCM, gray-level cooccurrence matrix.

Figure S1

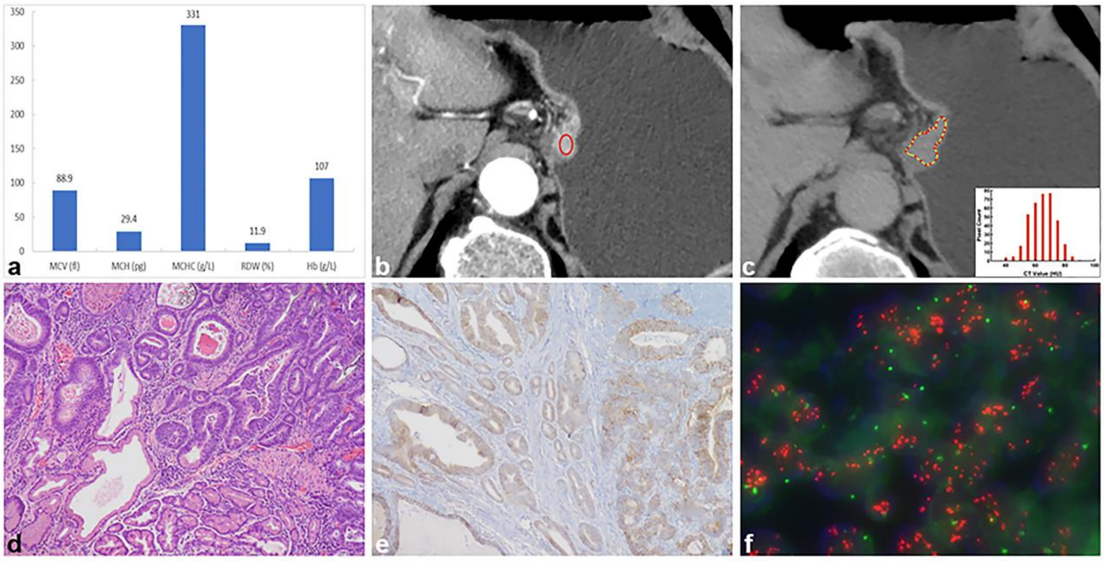

Figure S1 A 74-year-old man with gastric cancer pathologically diagnosed as human epidermal growth factor receptor 2 (HER2) positive. (a) The values of erythrocyte indices, including MCV, MCH, MCHC, RDW and Hb, were 88.9 fL, 29.4 pg, 331.0 g/L, 11.9% and 107.0 g/L, respectively. (b) Computed tomography (CT) image based on arterial phase shows a mass lesion with marked enhancement in the lesser curvature of the stomach body. An oval region of interest (ROI) was drawn to encompass the area of greatest enhancement on the maximal section, and the CT value-related parameters were extracted. (c) A polygonal ROI was manually drawn along the margin of the tumor on the largest cross-section, and the texture parameters were extracted. (d) Hematoxylin and eosin staining of a postoperative specimen shows a moderate/well differentiated carcinoma with a Lauren classification of intestinal type (original magnification, ×100). (e) HER2 immunohistochemistry shows moderate/weak complete or basolateral membranous staining in ≥10% of the cells (score 2+), (original magnification, ×100). (f) Fluorescent in situ hybridization test shows the ratio of HER2 gene (stained red) to chromosome 17 (stained green) signals is >2, which indicates HER2 gene amplification.

MCV, mean corpuscular volume; MCH, mean corpuscular hemoglobin; MCHC, mean corpuscular hemoglobin concentration; RDW, red cell distribution width; Hb, hemoglobin.
